# Supplementary material for: Characterization of an alternative BAK-binding site for BH3 peptides
Source: Nat Commun. 2020 Jul 3;11:3301. doi: 10.1038/s41467-020-17074-y (PMC7335050; doi:10.1038/s41467-020-17074-y)
Supplement: Supplementary file 1 — Supplementary Information [file 41467_2020_17074_MOESM1_ESM.pdf]

SUPPLEMENTARY INFORMATION FOR:

Characterization of an Alternative BAK Binding Site for BH3 Peptides

Kaiqin Ye[1,2], Wei X. Meng[3,4], Hongbin Sun[5], Bo Wu[6], Meng Chen[1,2], Yuan-Ping Pang[4], Jia Gao[1,2], Hongzhi Wang[1,2], Junfeng Wang[6], Scott H. Kaufmann[3,4,*] and Haiming Dai[1,2,*]

Affiliations:
[1]Anhui Province Key Laboratory of Medical Physics and Technology, Center of Medical Physics and Technology, Hefei Institutes of Physical Science, Chinese Academy of Sciences, Hefei, China
[2]Hefei Cancer Hospital, Chinese Academy of Sciences, Hefei, China
[3]Division of Oncology Research, Mayo Clinic, Rochester, MN
[4]Department of Molecular Pharmacology and Experimental Therapeutics, Mayo Clinic, Rochester, MN
[5]School of Food and Biological Engineering, Zhenzhou University of Light Industry, Zhenzhou, China
[6]High Magnetic Field Laboratory, Chinese Academy of Sciences, Hefei, China

*, co-corresponding authors: Haiming Dai, E-mail: daih@cmpt.ac.cn
or Scott H. Kaufmann, E-mail: Kaufmann.Scott@mayo.edu

CONTENTS:

Supplementary Figures 1-8.
Supplementary Tables 1-2.

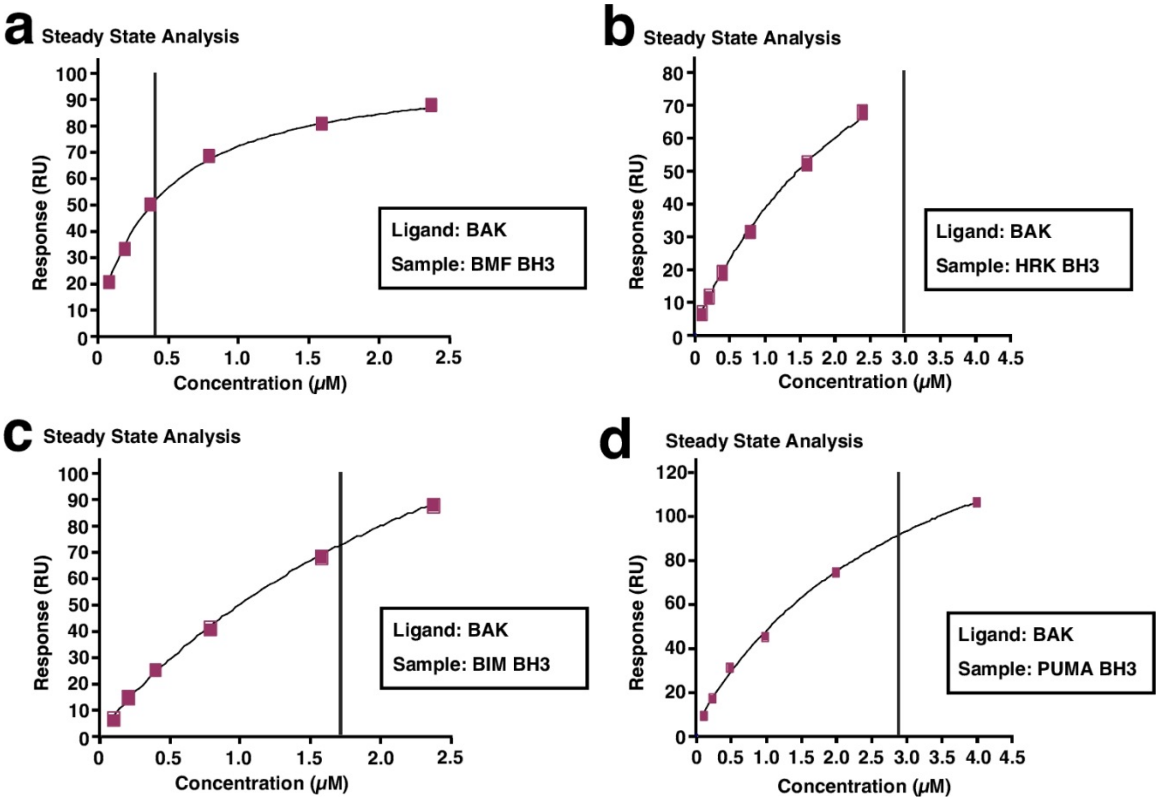

Supplementary Figure 1 Steady State Analysis of SPR assays shown in Figure 1. **a, b, c, d** Steady State Analysis of a representative SPR assay from three independent experiments for BAK binding to BMF BH3 (**a**), HRK BH3 (**b**), BIM BH3 (**c**), and PUMA BH3 (**d**), respectively.

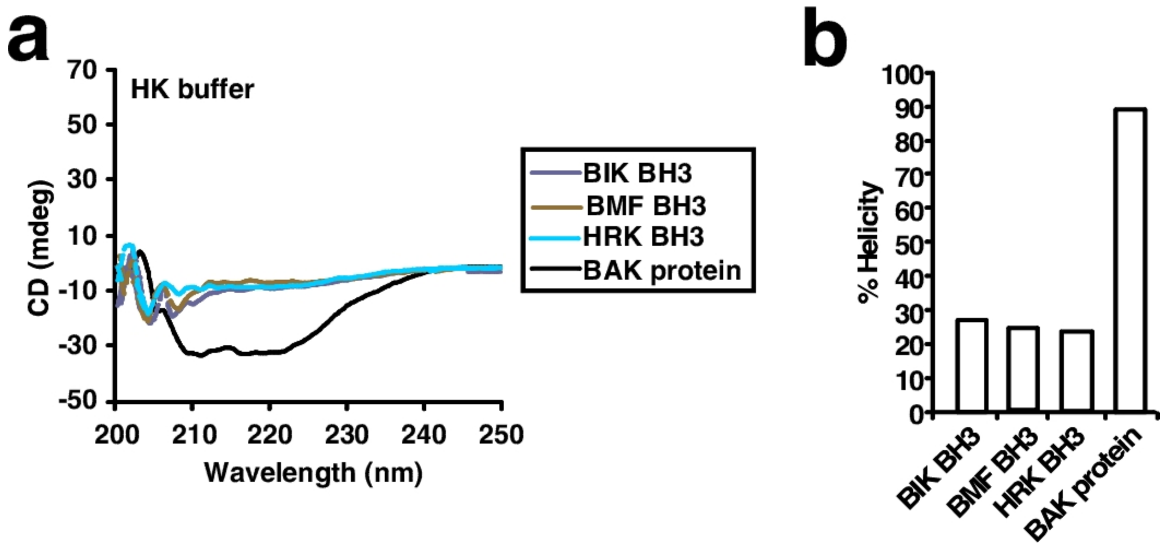

Supplementary Figure 2 Circular dichroism (CD) spectra of BH3 peptides used in Figure 1. **a** Far UV-CD spectra of indicated BH3 peptides (0.2 mg ml^{-1}) were measured in HS buffer. BAKΔTM served as a positive control of α-helical protein here. **b** α-helical content of BH3 domains calculated from CD experiments.

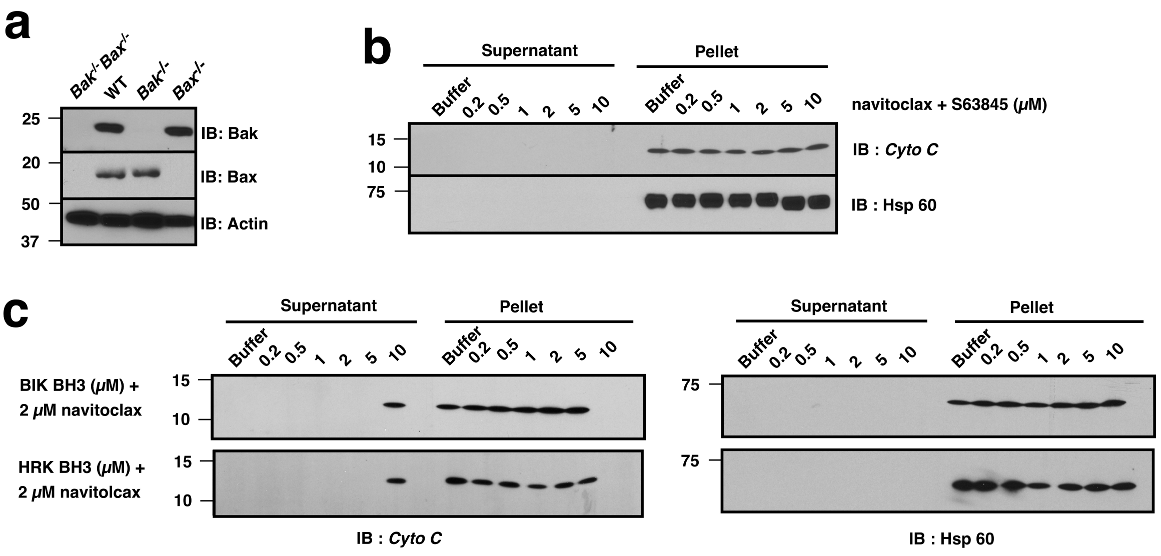

Supplementary Figure 3 Combination of navitoclax and S63845 does not induce *cytochrome c* release from mitochondria of *Bax*$^{-/-}$ MEFs. **a** *Bak* and/or *Bax* knock-out MEFs confirmed by western blotting. Cell lysates from the indicated MEFs cells (*Bax*$^{-/-}$ *Bak*$^{-/-}$, WT, *Bak*$^{-/-}$, and *Bax*$^{-/-}$) were subjected to SDS-PAGE and western blotting for Bak, Bax and β-actin. **b, c** After mitochondria from *Bax*$^{-/-}$ MEFs were incubated for 90 min at 25 °C with the indicated concentrations of navitoclax and S63845 (**b**) or combination of indicated BH3 peptides and inhibitors (**c**), supernatants and pellets were subjected to SDS-PAGE and immunoblotting for *cytochrome c* (*Cyto c*) and Hsp60.

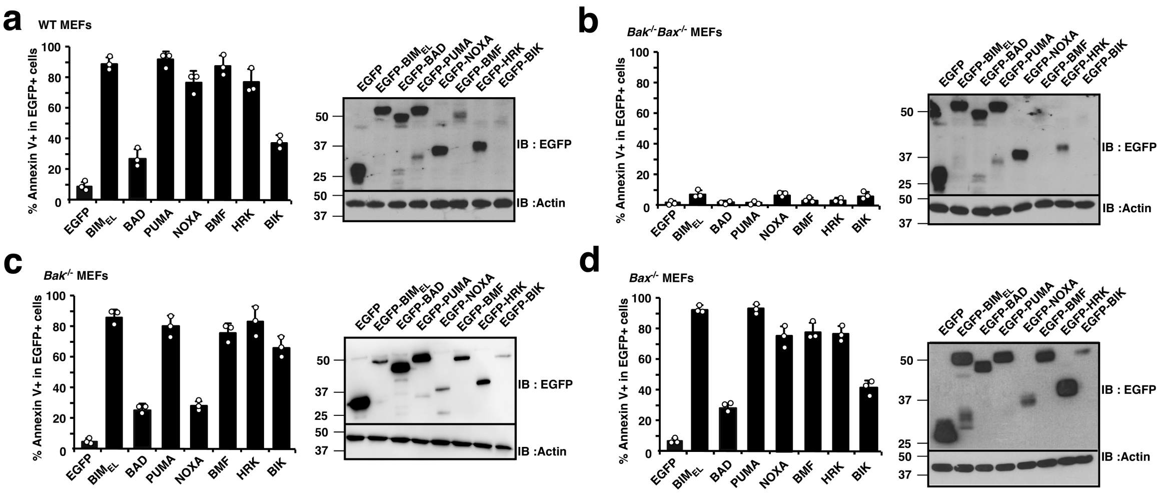

Supplementary Figure 4 BMF and HRK induce Bak/Bax-dependent apoptosis in MEFs. **a, b, c, d** After cDNAs encoding the indicated BH3-only proteins fused at their N termini to EGFP were transfected into WT (**a**), *Bax*$^{-/-}$*Bak*$^{-/-}$ (**b**), *Bak*$^{-/-}$ (**c**), or *Bax*$^{-/-}$ (**d**) MEFs for 24 h, cells were harvested, stained with APC-conjugated Annexin V, and subjected to flow cytometry. The percentages EGFP^{+} cells that were Annexin V^{+} are indicated. **Insets in panels a, b, c and d,** whole cell lysates subjected to immunoblotting. Error bars: mean ± S.D. of three independent experiments.

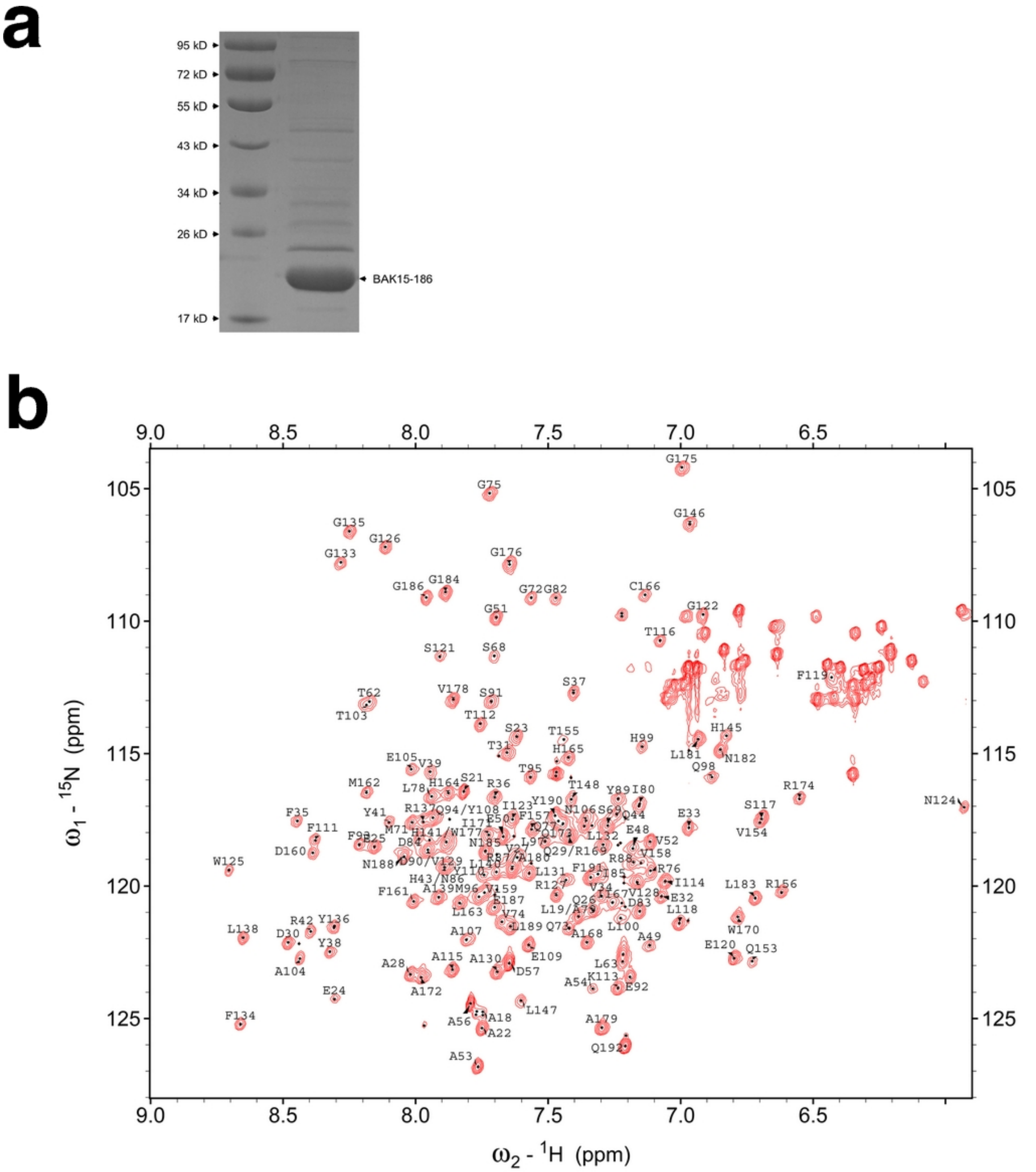

Supplementary Figure 5 Chemical shift assignments of BAK 15-186. **a** Purified BAK 15-186 for NMR study. **b** Using the 2D and 3D spectra described in Materials and Methods, chemical shifts of BAK residues 15-186 were assigned as shown on the ^{1}H, ^{15}N-HSQC spectra.

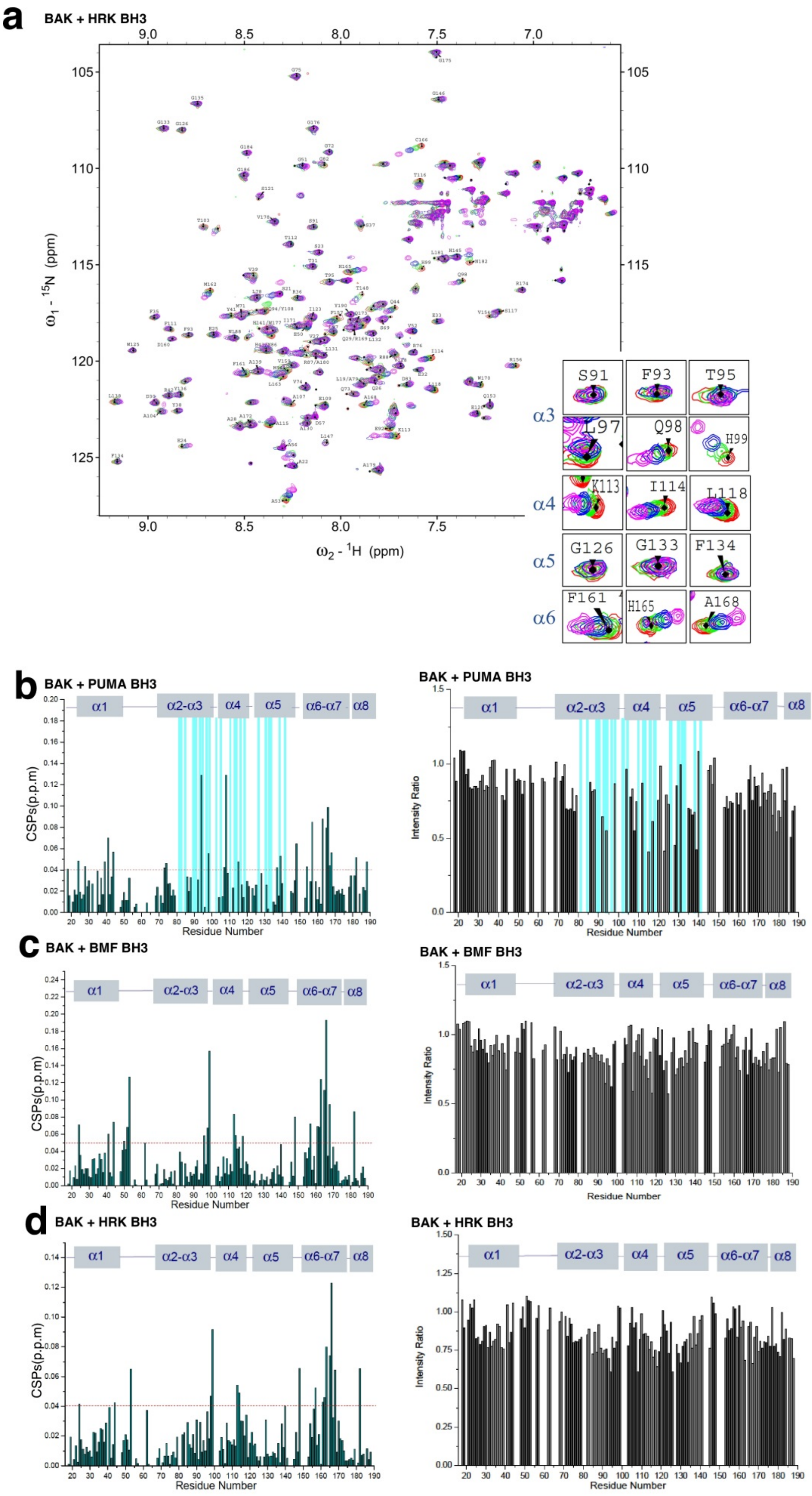

a
BAK + HRK BH3
ω1 - 15N (ppm)
ω2 - 1H (ppm)
α3
S91
F93
T95
L97
Q98
H99
α4
K113
I114
L118
α5
G126
G133
F134
α6
F161
H165
A168
b
BAK + PUMA BH3
CSPs(p.p.m)
Residue Number
Intensity Ratio
α1
α2-α3
α4
α5
α6-α7
α8
c
BAK + BMF BH3
d
BAK + HRK BH3

Supplementary Figure 6 Chemical shift changes induced by PUMA, BMF and HRK BH3s in $^{1}H,^{15}N$-HSQC spectrum of BAK 15-186. **a** $^{1}H,^{15}N$-HSQC spectra of 0.5 mM ^{15}N-labeled BAK 15-186 perturbed by different concentration ratios of HRK BH3 peptide as shown in different colors. Protein: peptide ratios were as follows: Red, no peptides; green, 1: 1; blue, 1: 2; and purple, 1:4. Peaks on the right indicate the residues that exhibit perturbations for comparison. **b-d** Chemical shift perturbations (CSPs, left) and Intensity Ratio (right) were plotted as a function for BAK residues, when $^{1}H,^{15}N$-HSQC spectra of BAK were recorded after titration of PUMA BH3 (**b**), BMF BH3 (**c**) and HRK BH3 (**d**) at the ratio of protein: peptide=1:4. Left, CSPs per BAK residue were calculated using the formula $CSPs=[(\Delta\delta NH^2+\Delta\delta N^2/25)/2]^{1/2}$. The threshold value was defined as average CSPs value plus one standard deviation. Right, cross peak intensity changes per BAK residue were indicated. Residues involved in interaction were defined by peak lost (the peak intensity decreased to the level of noise), shown as light blue bars.

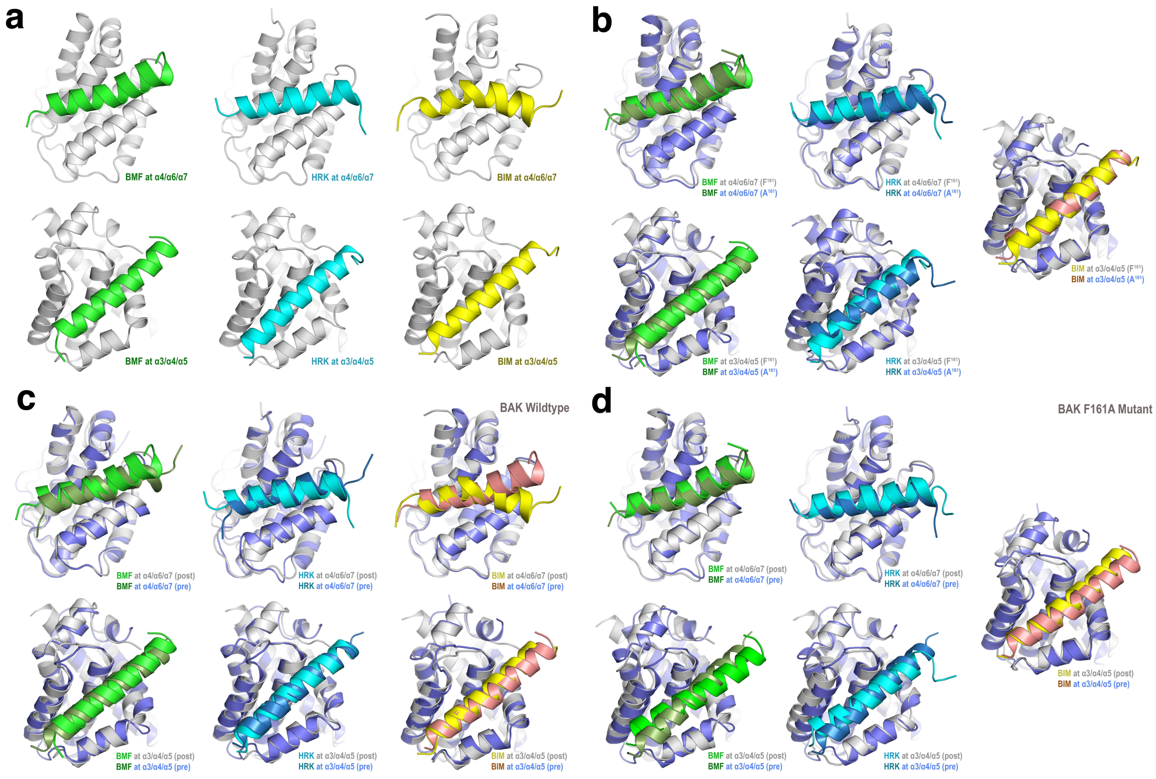

Supplementary Figure 7 Results of MD simulations. **a** Backbone conformations of BMF, HRK, and BIM binding at the alternative BH3-binding groove (α4, α6 and α7) or the canonical groove (α3, α4 and α5) of the wild-type BAK. Each conformation shown represents the respective top-1 cluster in Table S2 and was derived from 20 316-ns, distinct, independent, unrestricted, unbiased, and isobaric–isothermal MD simulations using FF12MC at 303 K (see Methods). These cartoon models show that BMF and HRK bind both grooves, whereas BIM binds the canonical groove only. **b** Overlays of the five complexes with high populations in panel a with the corresponding ones of the BAK F161A mutant. Each of the mutant complex conformations shown in the cartoon model also represents the respective top-1 cluster in Table S2 and was derived from 20 316-ns, distinct, independent, unrestricted, unbiased, and isobaric–isothermal MD simulations using FF12MC at 303 K (see Methods). These cartoon models show the contraction of the alternative groove and the expansion of the canonical groove in the mutant. The models also show that the F161A mutation changes the backbone conformations of BMF and HRK BH3 peptides but hardly that of BIM BH3 peptide. **c** Overlays of all six complexes in panel a with the corresponding ones that were used as the initial conformations for the MD simulations. These overlays shown in the cartoon model reveal backbone conformational changes of all three peptides. **d** Overlays of all five mutant complexes in panel b with the corresponding ones that were used as the initial conformations for the MD simulations. The overlays shown in the cartoon model reveal 1) backbone conformational changes of all three peptides, 2) the contraction of the alternative groove and the expansion of the canonical groove in the mutant when binding to BMF and HRK, and 3) hardly any contraction or expansion when binding to BIM. Here the contraction and expansion observed after the MD simulations were due to

the use of the initial conformations that were derived directly from the wild-type BAK complexes.

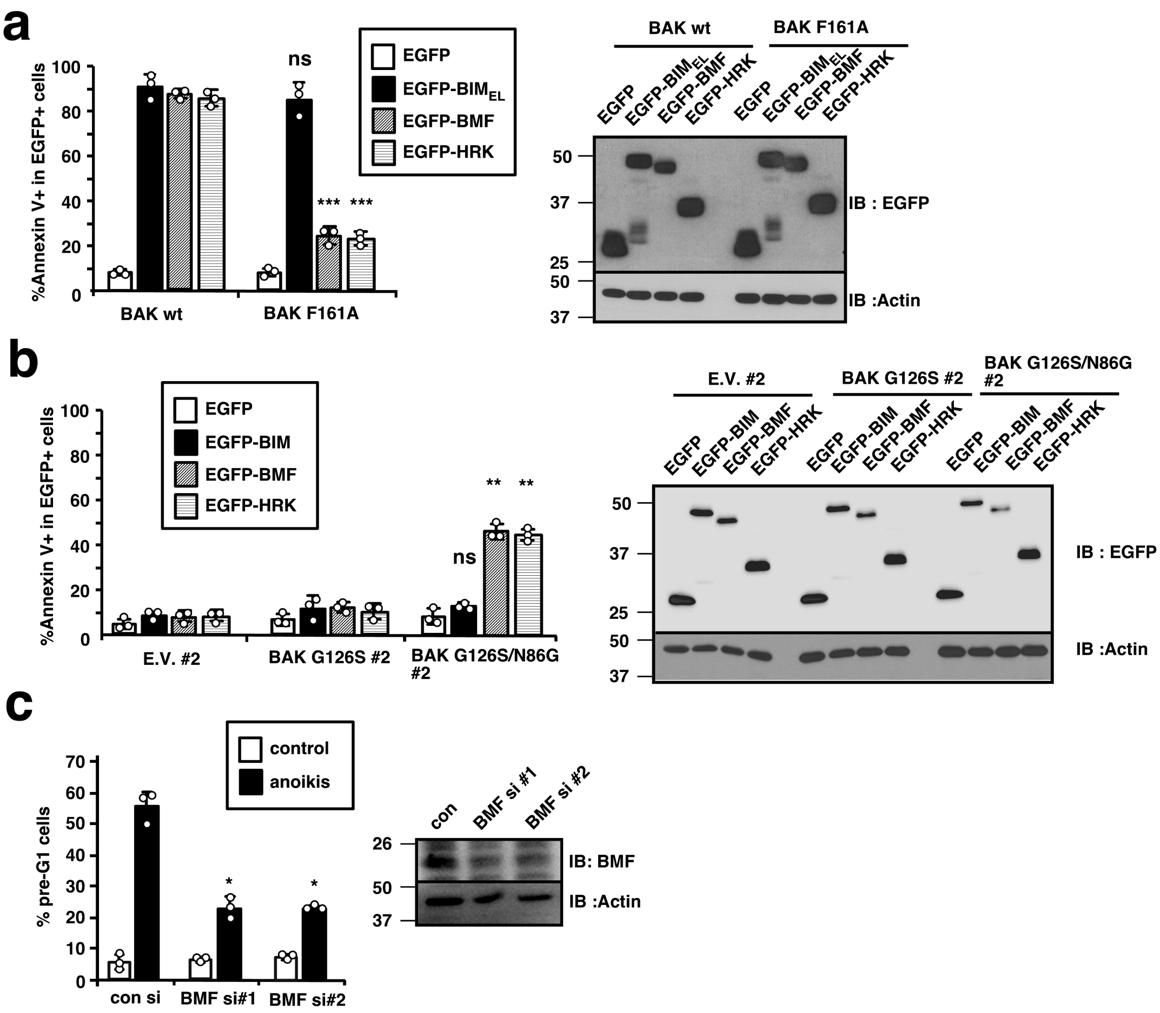

Supplementary Figure 8 BAK $\alpha6$ mutation inhibits BMF and HRK-induced BAK activation. **a, b** After the indicated EGFP-tagged BH3-only proteins were transfected into *Bax$^{-/-}$Bak$^{-/-}$* DKO MEFs reconstituted with wild type (WT) BAK, F161A BAK (**a**), or empty vector (E.V.), or BAK G126S without or with the reciprocal N86G mutation that restores BAK oligomerization (**b**), cells were harvested, stained with APC-conjugated Annexin V, and subjected to flow cytometry. The percentages of EGFP^{+} cells that are Annexin V^{+} are indicated. p=0.41, 0.0004. 2.8E-07 for BIM, BMF, and HRK, respectively when the percentage of Annexin V^{+} cells reconstituted with BAK F161A were compared to those reconstituted with WT BAK (**a**), and p=0.11, 0.009, 0.007 for BIM, BMF and HRK, respectively when the percentage of Annexin V^{+} cells reconstituted with BAK G126S/N86G was compared to thosed reconstituted with WT BAK (**b**). **c** After *Bax$^{-/-}$* MEFs were transfected with indicated BMF siRNAs for 24 h, cells were subjected to anoikis for another 48 h, followed by the analysis of the percentage of pre-G1 cells. p=0.02, 0.01 for BMF si#1 and BMF si#2, respectively when the percentage of pre-G1 cells was compared to con siRNA group induced by anoikis. **Right panels**, whole cell lysates subjected to immunoblotting. Error bars: mean ± S.D. of three independent experiments. ns, p>0.05, *, p<0.05; **, p<0.01, ***, p<0.001, two tailed paired t-test.

Supplementary Table 1. Chemical shift changes of amino acids involved in interaction between BAK and BH3 peptides

Assigment[a]			PUMA			BMF			HRK		
Amino acid	N	NH	N	NH	CSP[b]	N	NH	CSP	N	NH	CSP
E24N-NH	124.440	8.824	124.324[c]	8.777	0.048	124.332	8.754	0.071	124.374	8.783	0.042
A28N-NH	123.369	8.522	123.709	8.549	0.043	123.565	8.521	0.020	123.497	8.522	0.013
V39N-NH	115.565	8.454	116.040	8.449	0.048	115.941	8.455	0.038	115.851	8.455	0.029
Y41N-NH	117.647	8.568	disappear			117.751	8.627	0.060	117.687	8.607	0.039
Q44N-NH	117.243	7.719	117.491	7.770	0.057	117.783	7.770	0.074	117.437	7.757	0.043
V52N-NH	118.485	7.627	118.443	7.616	0.012	118.614	7.694	0.068	118.431	7.636	0.010
A53N-NH	127.225	8.283	126.933	8.269	0.032	126.277	8.199	0.127	126.690	8.246	0.065
G82N-NH	109.829	8.085	disappear			109.843	8.124	0.039	109.686	8.100	0.021
D83N-NH	121.244	7.657	disappear			121.152	7.631	0.028	121.169	7.636	0.022
I85N-NH	120.072	7.855	disappear			119.848	7.867	0.025	119.784	7.851	0.029
Y89N-NH	117.061	7.781	disappear			117.015	7.791	0.011	116.992	7.776	0.009
D90N-NH	118.794	8.486	disappear			118.777	8.473	0.013	118.664	8.458	0.031
S91N-NH	113.039	8.141	disappear			113.068	8.163	0.022	113.024	8.145	0.004
F93N-NH	118.670	8.790	disappear			118.658	8.783	0.007	118.601	8.784	0.009
Q94N-NH	117.419	8.395	disappear			117.453	8.409	0.014	117.442	8.412	0.017
T95N-NH	115.872	8.058	disappear			115.983	8.050	0.014	115.951	8.052	0.010
M96N-NH	120.487	8.276	disappear			120.791	8.326	0.059	120.679	8.307	0.036
L97N-NH	118.559	8.037	disappear			118.403	8.056	0.025	118.386	8.043	0.018
Q98N-NH	115.830	7.368	116.224	7.407	0.055	115.969	7.434	0.067	115.962	7.413	0.047
H99N-NH	115.205	7.578	disappear			114.331	7.708	0.157	114.651	7.651	0.092
T103N-NH	113.016	8.713	disappear			113.102	8.721	0.012	113.115	8.714	0.010
E105N-NH	115.548	8.475	disappear			115.495	8.476	0.005	115.547	8.486	0.011
Y110N-NH	119.511	8.299	disappear			119.443	8.288	0.013	119.437	8.287	0.014
F111N-NH	118.368	8.883	disappear			118.335	8.908	0.025	118.292	8.899	0.018
K113N-NH	123.915	7.711	disappear			123.842	7.794	0.083	123.857	7.765	0.054
I114N-NH	119.835	7.532	disappear			120.008	7.588	0.059	119.937	7.580	0.049
A115N-NH	123.328	8.367	122.873	8.381	0.048	122.979	8.342	0.043	123.066	8.352	0.030
T116N-NH	110.664	7.589	disappear			111.095	7.603	0.045	110.961	7.593	0.030
L118N-NH	121.517	7.512	disappear			121.378	7.568	0.058	121.380	7.543	0.034
G122N-NH	109.900	7.433	disappear			109.825	7.423	0.013	109.835	7.421	0.014
G126N-NH	108.010	8.823	disappear			108.014	8.821	0.002	107.993	8.817	0.006
A130N-NH	123.220	8.173	disappear			123.207	8.180	0.007	123.257	8.180	0.008
L132N-NH	118.550	7.831	disappear			118.539	7.821	0.010	118.576	7.837	0.007
G133N-N	107.915	8.919	disappear			107.908	8.918	0.001	107.909	8.910	0.009
F134N-NH	125.228	9.159	disappear			125.170	9.154	0.008	125.206	9.151	0.008
A139N-NH	120.555	8.429	120.411	8.378	0.053	120.487	8.418	0.013	120.500	8.418	0.012
H141N-NH	118.337	8.382	disappear			118.265	8.389	0.010	118.383	8.380	0.005
F157N-NH	117.804	8.020	117.984	8.103	0.085	118.221	8.079	0.072	118.091	8.064	0.053
V158N-NH	119.098	7.653	disappear			119.276	7.657	0.018	119.217	7.660	0.014
F161N-NH	120.690	8.492	120.517	8.508	0.024	120.619	8.561	0.069	120.605	8.534	0.043

M162N-NH	116.344	8.677	disappear			116.848	8.723	0.068	116.703	8.706	0.046
L163N-NH	120.807	8.298	120.781	8.386	0.088	120.791	8.326	0.124	120.598	8.375	0.080
H165N-NH	115.383	7.950	115.190	7.873	0.079	115.209	7.840	0.111	115.214	7.878	0.074
C166N-NH	108.839	7.580	109.179	7.673	0.099	109.462	7.763	0.193	109.246	7.696	0.123
A168N-NH	122.276	7.879	122.279	7.823	0.056	122.056	7.787	0.095	122.139	7.816	0.064

a: 146 residues out of 171 residues of BAK^{1}H-^{15}N HSQC were assigned (18-188, including 6 pro that do not have peaks).
b: The chemical shift perturbation was calculated by the formula CSP=$[(\Delta\delta_{NH}{}^{2}+\Delta\delta_{N}{}^{2}/25)/2]^{1/2}$.
c: Dark background: Amino acids with significant chemical shift changes. Pink background: Amino acids disappeared.

Supplementary Table 2. Cluster analysis of MD simulations of CL025 folding as a control and BAK and its F161A mutant in complex with BH3 peptides

Temperature (K)	Number of conformers	Number of conformers in each of the top-3 clusters		Occurrence of each cluster (%)		CRMSD* (Å)
		BMF at the noncanonical site				
		Wild-type	F161A	Wild-type	F161A	
		13,751	6,304	69	32	NA$^{\#}$
303	20,000	912	4,551	5	23	NA$^{\#}$
		645	2,819	3	14	NA$^{\#}$
		BMF at the canonical site				
		Wild-type	F161A	Wild-type	F161A	
		12,891	4,488	65	22	NA$^{\#}$
303	20,000	1,459	3,206	7	16	NA$^{\#}$
		954	2,759	5	14	NA$^{\#}$
		HRK at the noncanonical site				
		Wild-type	F161A	Wild-type	F161A	
		10,660	4,357	53	22	NA$^{\#}$
303	20,000	1,750	1,415	9	7	NA$^{\#}$
		749	1,127	4	6	NA$^{\#}$
		HRK at the canonical site				
		Wild-type	F161A	Wild-type	F161A	
		14,712	6,353	74	32	NA$^{\#}$
303	20,000	1,082	6,205	5	31	NA$^{\#}$
		949	3,746	5	19	NA$^{\#}$
		BIM at the noncanonical site				
		Wild-type		Wild-type		
		7,056		35		NA$^{\#}$
303	20,000	2,461		12		NA$^{\#}$
		1,307		7		NA$^{\#}$
		BIM at the canonical site				
		Wild-type	F161A	Wild-type	F161A	
		14,988	9,983	75	50	NA$^{\#}$
303	20,000	1,371	2,265	7	11	NA$^{\#}$
		1,159	2,182	6	11	NA$^{\#}$
		CLN025 folding (control)				
		13,072		65		1.7
300	20,000	1,352		7		4.7
		818		4		4.8
		CLN025 folding (control)				
		14,092		71		1.7
277	20,000	894		5		4.8
		621		3		4.8

CRMSD*: Alpha carbon root mean square deviation of the average conformer in each cluster from the experimentally determined structure. NA$^{\#}$: Not available due to the lack of the experimentally determined structure.
