# Supplementary material for: Characterization of an alternative BAK-binding site for BH3 peptides
Source: Nat Commun. 2020 Jul 3;11:3301. doi: 10.1038/s41467-020-17074-y (PMC7335050; doi:10.1038/s41467-020-17074-y)
Supplement: Supplementary file 3 — Reporting Summary [file 41467_2020_17074_MOESM3_ESM.pdf]

nature research

Corresponding author(s): Haiming Dai and Scott Kaufmann

Last updated by author(s): May 24, 2020

Reporting Summary

Nature Research wishes to improve the reproducibility of the work that we publish. This form provides structure for consistency and transparency in reporting. For further information on Nature Research policies, see our Editorial Policies and the Editorial Policy Checklist.

Statistics

For all statistical analyses, confirm that the following items are present in the figure legend, table legend, main text, or Methods section.

n/a	Confirmed	
☐	☒	The exact sample size (n) for each experimental group/condition, given as a discrete number and unit of measurement
☐	☒	A statement on whether measurements were taken from distinct samples or whether the same sample was measured repeatedly
☐	☒	The statistical test(s) used AND whether they are one- or two-sided<br>*Only common tests should be described solely by name; describe more complex techniques in the Methods section.*
☒	☐	A description of all covariates tested
☒	☐	A description of any assumptions or corrections, such as tests of normality and adjustment for multiple comparisons
☐	☒	A full description of the statistical parameters including central tendency (e.g. means) or other basic estimates (e.g. regression coefficient) AND variation (e.g. standard deviation) or associated estimates of uncertainty (e.g. confidence intervals)
☐	☒	For null hypothesis testing, the test statistic (e.g. F, t, r) with confidence intervals, effect sizes, degrees of freedom and P value noted<br>*Give P values as exact values whenever suitable.*
☒	☐	For Bayesian analysis, information on the choice of priors and Markov chain Monte Carlo settings
☒	☐	For hierarchical and complex designs, identification of the appropriate level for tests and full reporting of outcomes
☒	☐	Estimates of effect sizes (e.g. Cohen's d, Pearson's r), indicating how they were calculated

Our web collection on statistics for biologists contains articles on many of the points above.

Software and code

Policy information about availability of computer code

Data collection	Flow cytometry (CytExpert_1.2.11), NMR (Topspin), Liposome release (SoftMax Pro 7.0.3), Biacore (Biacore T200 Control Software), Simulation (AmberTools 13 and 16)
Data analysis	Flow cytometry (CytExpert_1.2.11), NMR (NMRPipe, Sparky 3), Statistics (Microsoft Excel 2007), Biacore (Biacore T200 Evaluation Software 3.1), Secondary Structure (k2d2 algorithm), Simulation (AmberTools 13 and 16)

For manuscripts utilizing custom algorithms or software that are central to the research but not yet described in published literature, software must be made available to editors and reviewers. We strongly encourage code deposition in a community repository (e.g. GitHub). See the Nature Research guidelines for submitting code & software for further information.

Data

Policy information about availability of data

All manuscripts must include a data availability statement. This statement should provide the following information, where applicable:

- Accession codes, unique identifiers, or web links for publicly available datasets
- A list of figures that have associated raw data
- A description of any restrictions on data availability

The data supporting the findings of this study are available in the manuscript and supplementary files or are available from the corresponding author upon request. The source data underlying Figures 1f, 2b-g, 3b-3i, 6b, 6d,6 g, 7b-g and Supplementary Figures 3b-c, 4a-d, 8a-c, are provided as a Source Data file.

Field-specific reporting

Please select the one below that is the best fit for your research. If you are not sure, read the appropriate sections before making your selection.

- [x] Life sciences
- [ ] Behavioural & social sciences
- [ ] Ecological, evolutionary & environmental sciences

For a reference copy of the document with all sections, see nature.com/documents/nr-reporting-summary-flat.pdf

Life sciences study design

All studies must disclose on these points even when the disclosure is negative.

Sample size	No statistical method was used to predetermine sample size. Sample sizes were chosen based an attempt to balance the error in estimating confidence intervals with the cost of replicating experiments.
Data exclusions	No data were excluded.
Replication	At least 3 independent experiments were performed for flow cytometry, surface plasmon resonance and liposome release assays and the findings were reliably produced. At least 2 independent experiments for immunoblots and immunoprecipitation assays were performed. Please refer to methods section for details.
Randomization	Samples were randomly allocated into experimental groups prior to treatment.
Blinding	One individual performed the experiment while another individual (blinded to the group allocation) performed the analysis. Investigators were blinded to group allocation during data collection.

Reporting for specific materials, systems and methods

We require information from authors about some types of materials, experimental systems and methods used in many studies. Here, indicate whether each material, system or method listed is relevant to your study. If you are not sure if a list item applies to your research, read the appropriate section before selecting a response.

Materials & experimental systems

n/a	Involved in the study	
[ ]	[x]	Antibodies
[ ]	[x]	Eukaryotic cell lines
[x]	[ ]	Palaeontology and archaeology
[x]	[ ]	Animals and other organisms
[x]	[ ]	Human research participants
[x]	[ ]	Clinical data
[x]	[ ]	Dual use research of concern

Methods

n/a	Involved in the study	
[x]	[ ]	ChIP-seq
[ ]	[x]	Flow cytometry
[x]	[ ]	MRI-based neuroimaging

Antibodies

Antibodies used	The following antibodies were used at 1/1000 dilutions for western blotting: Hsp60 (D307, cat. #4870S), EGFP (#2555S), BAX (#2772S), BIM (#2933S), BID (#2002S), BCLxL(#2764S), MCL1 (#4572S) from Cell Signaling Technology; cytochrome c (#556433) from BD Biosciences; BAK N-term antibody (#06-536) and BAK Ab-1 antibody (TC-100) from Millipore; BCL2 antibody from DAKO (#M0887); NOXA antibody from Enzo Life Sciences (#ALX-804-408-c100); BMF antibody from Proteintech (#18298-1-AP); and actin (goat polyclonal, I-19, #sc-1615) and PUMA (#sc-374223) from Santa Cruz Biotechnology. Anti-S peptide antibody was raised in our laboratory and has been validated.
Validation	Validation was based on information provided in manufacturers' datasheets, except the anti-S peptide antibody, which was validated by our lab (see reference):<br>Hsp60, https://www.cellsignal.com/products/primary-antibodies/hsp60-d307-antibody/4870<br>EGFP, https://www.cellsignal.com/products/primary-antibodies/gfp-antibody/2555<br>BAX, https://www.cellsignal.com/products/primary-antibodies/bax-antibody/2772<br>BIM, https://www.cellsignal.com/products/primary-antibodies/bim-c34c5-rabbit-mab/2933<br>BID, https://www.cellsignal.com/products/primary-antibodies/bid-antibody-human-specific/2002<br>BCLXL, https://www.cellsignal.com/products/primary-antibodies/bcl-xl-54h6-rabbit-mab/2764<br>MCL1, https://www.cellsignal.com/products/primary-antibodies/mcl-1-antibody/4572<br>Cytochrome C, https://www.bdbiosciences.com/us/applications/research/apoptosis/purified-antibodies/purified-mouse-anti-cytochrome-c-7h82c12/p/556433<br>BAK N-term, https://www.sigmaaldrich.com/catalog/product/mm/06536?lang=en&region=US<br>BAK Ab-1, https://www.emdmillipore.com/US/en/product/Anti-Bak-Ab-1-Mouse-mAb-TC-100,EMD_BIO-AM03

BCL2, https://www.agilent.com/store/productDetail.jsp?catalogId=M088701-2
NOXA, https://www.enzolifesciences.com/ALX-804-408/noxa-monoclonal-antibody-114c307.1/
BMF, https://www.ptglab.com/Products/BMF-Antibody-18298-1-AP.htm
Actin, https://www.scbt.com/p/actin-antibody-c-11?requestFrom=search
PUMA, https://datasheets.scbt.com/sc-374223.pdf
S-peptide, https://pubmed.ncbi.nlm.nih.gov/15560139/

Eukaryotic cell lines

Policy information about cell lines

Cell line source(s)	Jurkat (Paul Leibson, Mayo Clinic). MEFs: wt, Bax-/-, Bak-/-, double knockout (Andrew Badley, Mayo Clinic). HEK293T (Richard Bram, Mayo Clinic).
Authentication	Jurkat and HEK293T were authenticated by STR assay in the Mayo Clinic Cytogenetics Laboratory. MEFs were validated by immunoblotting for the indicated proteins and failure to react with HSP90 antibody specific for the human protein.
Mycoplasma contamination	All cell lines used in this study were routinely tested for mycoplasma and found to be mycoplasma free.
Commonly misidentified lines (See ICLAC register)	No commonly misidentified lines were used in the study.

Flow Cytometry

Plots

Confirm that:

- [x] The axis labels state the marker and fluorochrome used (e.g. CD4-FITC).
- [x] The axis scales are clearly visible. Include numbers along axes only for bottom left plot of group (a 'group' is an analysis of identical markers).
- [ ] All plots are contour plots with outliers or pseudocolor plots.
- [x] A numerical value for number of cells or percentage (with statistics) is provided.

Methodology

Sample preparation	A citation describing sample preparation is included in the Methods section.
Instrument	Beckman Coulter CytoFLEX was used for data collecting and its Model No. was A00-1-1102.
Software	CytExpert version 1.2
Cell population abundance	No sorting was conducted.
Gating strategy	Analysis was performed without gating. Virtually all cells were included for further evaluation. An example of the data generated is shown in Figure 3a.

- [ ] Tick this box to confirm that a figure exemplifying the gating strategy is provided in the Supplementary Information.
